# The Impact of Sustainable Growth and Sustainable Environment on Public Health: A Study of GCC Countries

**DOI:** 10.3389/fpubh.2022.887680

**Published:** 2022-03-31

**Authors:** Mohd Naved Khan, Ghazala Aziz, Mohd Saeed Khan

**Affiliations:** ^1^Department of Business Administration, College of Administrative and Financial Sciences, Saudi Electronic University, Riyadh, Saudi Arabia; ^2^Finance and Economics Department, College of Business, University of Jeddah, Jeddah, Saudi Arabia

**Keywords:** public health, sustainable environment, sustainable growth, GCC, ARDL

## Abstract

The current study investigates the impact of economic growth, carbon emission, temperature, and environmental technology on public health in GCC countries. Panel data from 1990 to 2020 is used, and the panel unit root test is used to check the stationarity of the data. After cointegration analysis, the ARDL estimation technique checks the long-run and short-run association between variables. The results have proved that economic growth enhances exposure to PM2.5 and mortality but helps in increasing life expectancy. Likewise, carbon emission also enhances exposure to PM2.5 and mortality but improves life expectancy. As far as temperature is concerned, although it increases the exposure to PM2.5, it also increases life expectancy. It is also found that environmental technology enhances exposure to PM2.5. For policy implication, the study reports that investment in research and development and modifications the energy mix are key measures to enhance the public health in GCC countries.

## Introduction

**Figure 1:**
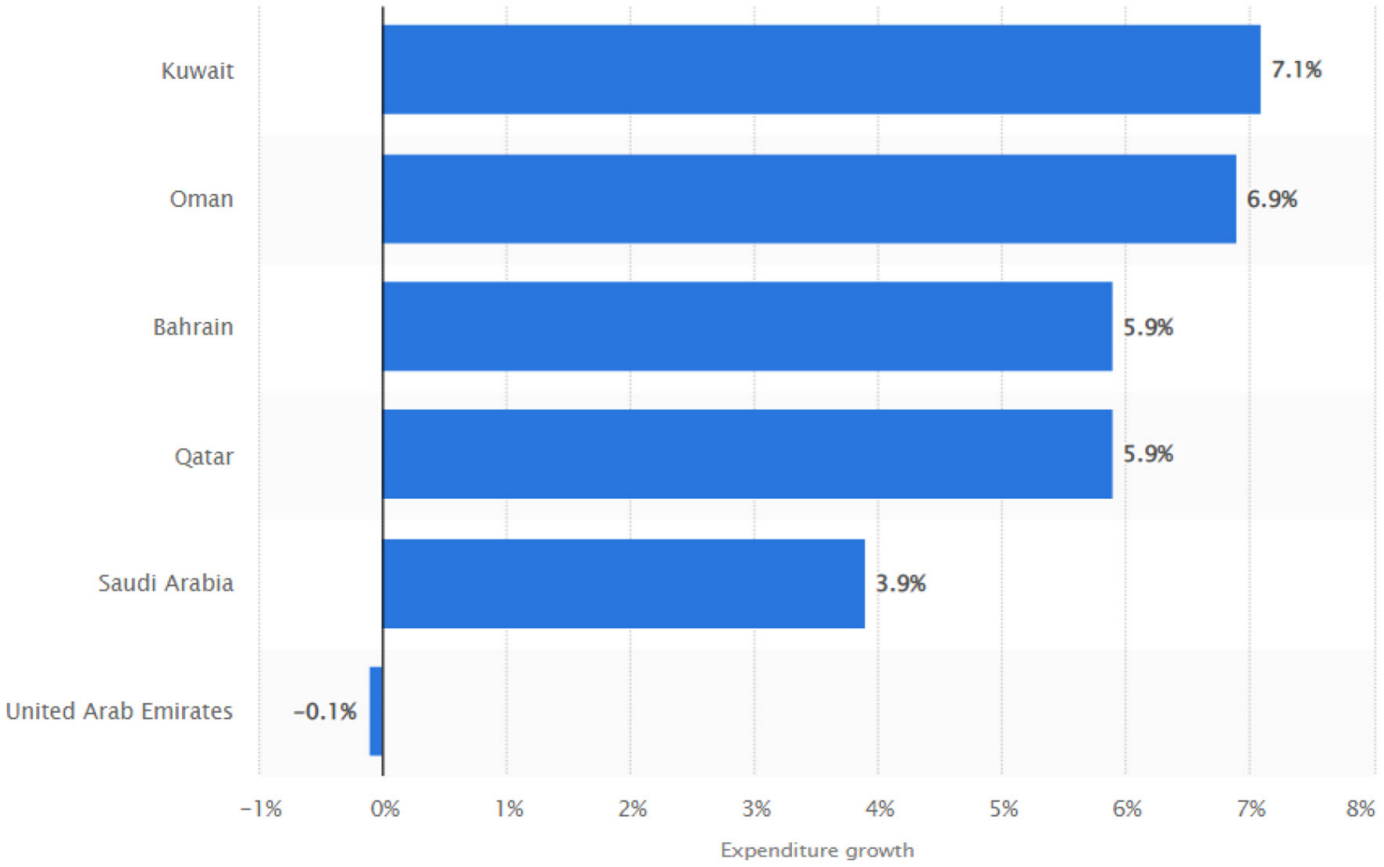
Distribution of healthcare expenditure growths in the Gulf Cooperation Council between 2010 and 2017, by country, 2022.

Increased temperature and carbon emission are major culprits behind most health issues in Gulf Corporation Council (GCC) countries (Bahrain, Iraq, Kuwait, Oman, Qatar, Saudi Arabia, and the United Arab Emirates). The reason is the abundance of oil and petroleum resources used for energy purposes in these countries. However, it has been proved that fossil fuel combustion is the major source of carbon emission. Also, the climate of this region is inherently hot, creating a vast majority of health-related issues. The issues due to carbon emission and a temperature range from skin irritation to lungs problem and even mortality due to exposure to harmful gasses and extreme temperature (1–3). GCC countries are spending a huge amount of money on health, and this can be seen in Figure 1, showing growth in health expenditure in these countries in recent years. This increase in expenditure can be attributed to health issues created by environmental degradation. To curb the harmful effects of environmental issues, spending on healthcare is very high in all countries, and environmental issues are the major reason behind this. Though, many initiatives are taken by these countries to diversify the economy from oil to non-oil sector (4). Still it is noted that due to global warming, the temperature in these states is expected to rise substantially in the coming year, which will create more health issues. This situation calls instant attention to this issue so that public health in this region can be protected in the coming future. Yet, the reasons behind public health issues and increased healthcare expenditure in GCC are neglected in the literature. Examining the determinants can help to introduce possible solutions; hence, it is essential to use empirical data and come up with practical solutions.

Previously researchers have tried to explore the environmental factors creating issues for public health (1–3, 5–7) and some practical solutions are also advised based on research (8–11) suggest that forests can play a significant role in improving public health. They assert that harmful gasses from the atmosphere are captured through trees, and also, forests can cool down the environment by attracting more rains. Also, Shuai et al. (12) found that technology and income level can play a vital role in enhancing public health by reducing carbon emissions. Sarwar et al. (13) and Sarwar et al. (14) have reported the nexus between economy, environment and public health.

Although researchers provide some solutions to improve public health, declining public health and increasing health expenditure are still present in GCC countries. The current study is an effort to check the determinants of public health so that important policy implications can be advised based on results. In this regard, there are four major contributions to this study; firstly, it is examining the impact of sustainable economic growth on public health in the GCC context. Economic growth can improve or decline public health through high-income levels and environmental degradation by the combustion of fossil fuels (15–18). The economies of GCC countries are dependent on the oil sector, and the abundance of oil resources makes it a major source of energy. This makes these economies grow through oil exports, as well to use non-renewable energy for public need which is responsible for higher emission in GCC countries. Resultantly, the public health degrades due to the high emission of greenhouse gasses. It has been proved that an excessive amount of carbon in the atmosphere destabilizes the environment, which adversely affects human health and increases hospitalization and deaths (19–22). Previously, few studied attempted to examine the nexus, however, as far our limited knowledge, the study is the pioneer which empirically investigated this nexus using GCC data.

The second contribution is exploring the association between environmental temperature and public health in GCC countries. It is a fact that these countries have hot climates compared to the rest of the world. But researchers argue that extreme weather, either hot or cold is dangerous for health (2, 6, 23). Extremely hot temperature results in multiple health issues, including skin diseases (2, 6, 23). However, it is still unknown how extreme temperature in GCC countries affects the public health. The current study takes the initiative and uses data from GCC countries to investigate the impact of temperature on public health.

The third contribution is filling the research gap in terms of the effect of environmental innovation on the public health of GCC countries. Although some researchers checked the significance of environmental technology on human health (24, 25), empirical studies lack in terms of GCC countries. To fill this gap, the study uses data from GCC countries and explores this relationship. Technical advancements in the environment can enhance health by reducing carbon emissions (26) and effectively managing environment-related data (25). GCC countries can also benefit from environmental technology in the reduction of health expenditure and enhancement in public health. However, empirical results are needed to put forward policy implications, and the current study is a forerunner in this sense.

Hence, based on the discussion above current study has four major objectives. First, to check the impact of economic growth on public health in GCC countries. Second to check the association between carbon emission and public health. Third to check the nexus between temperature and public health. The last objective is to investigate the importance of environment-related technology for public health in the GCC context.

## Literature Review

### Economic Growth and Public Health

Economic growth and the public go hand in hand. When the economy grows, it improves public health by providing better health facilities and high income to get healthy and nutritious food. Researchers try to explore this nexus, and in this regard Erdogan et al. (27) used data from 25 OECD countries from 1970 to 2007. Their analysis proved that economic growth and public health in terms of mortality rates are significantly and negatively related. Hence, they concluded that the mortality rate reduces when countries get rich and powerful.

Similarly, Lu et al. (28) used Chinese data from 2002 to 2014 to know if economic growth in China affects its population's health by reducing the mortality rate. This study shows that although China's economy is grown significantly, it is reducing the mortality rate in the country. In the same line, Pakistani data is explored by Wang et al. (22), who used data from 1995 to 2017 to investigate if the economic growth of Pakistan is beneficial for public health in a country. Using the ARDL approach, they found that economic growth enhances health expenditure. Hence, overall public health enhances ultimately. A recent study by Niu et al. (17) also found that the economic growth of China has a significant threshold effect on public health in China. They assert that improvement in public health is seen after the threshold.

### Carbon Emission and Public Health

Public health is also severely affected due to adverse environments, and high carbon emission is the major factor. Dong et al. (29) used provincial data from China from 2002 to 2017 to check if carbon emission affects public health. Their analysis proved that the long-term health impacts of carbon emissions are negative and that a major percentage of patients increases due to a 1% increase in carbon emissions. Another study by Wu et al. (30) used city-level data to explore the impact of carbon peaking on health outcomes. They found that reducing carbon emission is essential to improve public health. The high amount of carbon in the atmosphere increases public health expenditure due to higher health issues. Bi and Hansen (31) conducted research in Australia and found that health issues and health expenditures significantly increased because carbon emissions are increasing in the country. In the same line, Wang et al. (22) used Pakistani data from 1995 to 2017 and applied ARDL to check the association. Their results prove that public health is negatively affected due to the increased concentration of carbon in the atmosphere of Pakistan. Niu et al. (17) used quantile regression to check the nexus between carbon emission and public health in the Chinese context. They also assert that due to rapid economic growth and a high amount of fossil fuel consumption, the health of the population is severely affected due to carbon emission. Similarly, Chen et al. (32), Liu and Guo (33) and Zhao et al. (34) also addressed the relationship between carbon emission and public health.

### Temperature and Public Health

High temperature is also considered dangerous for health, and it increases the mortality rate as well. Ostro et al. (6) investigated this relationship from major countries from 1983 to 2006. They note that elevated temperature proved to be the major cause of deaths not only in the past but also in the future. Temperature can increase the mortality rate due to adverse effects on health. Also, Rauf et al. (7) used data from Pakistan to check the impact of excessive heat on the health of residents. Their analysis shows that heatwaves in Pakistan negatively affected the health of the country's residents. Another study by Campbell et al. (5) conducted a global systematic review to check the impact of severe temperature events on health outcomes. They found that a high mortality rate is associated with increased temperature. The effect of temperature is not only in terms of heat, but cold temperature also affects health similarly. To check this Gasparrini et al. (23) investigated data from different countries and found that cold temperature is more dangerous for health than hot temperature. Hence, hot or cold temperature leads to a high mortality rate. Additionally, Yang et al. (2) tried to explore the impact of temperature variability on different health-related issues. They proved that temperature played a vital role in different health outcomes and deaths due to disease.

### Technology and Public Health

Countries worldwide are struggling to mitigate the negative impacts of climate change and global warming on human health. It is noted that technical advancements in the environment enhance human health by reducing emissions and temperature. Researchers are trying to explore the significance of environment-related technology for public health and, in this context Hussain et al. (24), tried to check the implications of environment technology for healthcare in multiple countries. They assert that environmental expenditures improve public health; however, in this process, healthcare expenditure enhances. Another study by Comess et al. (25) also found that technical advancements in the environment improve public health. They suggest that data gathering and analysis can enhance the understanding of the environment externalities, which can be used to alter the improvements in public health. Jiang et al. (26) used data from BRICS economies to check the effect of green technology on health outcomes in these countries. Their results prove that life expectancy is significantly enhanced due to green technology.

In view of the previous literature, it appears that existing literature misses the context of GCC; some of the studies used the world level data, some for specific country or regions. Whereas, the nexus between sustainable economic growth, sustainable environment and public health, for GCC countries, is missing. Blair et al. (35) examined the dynamics of public health in Arab world, in theoretical perspective, instead of empirical examination. However, the current study is an attempt to investigate the missing gap which provide important policy implication to curb the public health issues.

## Data and Methodology

### Data

The current study aims to check the impact of economic growth, carbon emission, temperature, and environmental technology on public health. The annual data for GCC countries including Bahrain, Oman, Qatar, Kuwait, Saudi Arabia, UAE from 1990 to 2020 is used for analysis. The Organization for Economic Co-operation and Development (OECD) is used to collect the data, and definition along with information regarding data sources is presented in Table 1.

**Table 1:** Data source.

**Variables**	**Definition**	**Source**
Dep Var
EXPOSURE	Exposure to PM2.5	OECD
DEATH	Mortality from exposure to ambient PM2.5 (Per 1,000,000 inhabitants)	OECD
LIFE	Life expectancy at birth (years)	OECD
Indep Var
EG	Real GDP	OECD
CO_2_	CO_2_ emissions from air transport per capita (Tons)	OECD
TEMP	Annual surface temperature, change since 1951–1980 (Number)	OECD
TECH	Development of environment-related technologies, % all technologies (Percentage)	OECD

*Dep Var and Indep Var stand for dependent and independent variables, respectively. OECD is Organisation for Economic Co-operation and Development (source: https://data.oecd.org/)*.

The basic models of current study are as follows:

*Model 1*


(1) $$\begin{array}{r} Exposure=\;\beta_{0}+\beta_{1}EG+\beta_{2}CO_{2}+\beta_{3}TEMP+\beta_{4}TECH+\varepsilon_{it} \end{array}$$

*Model 2*


(2) $$\begin{array}{r} DEATH=\;\beta_{0}+\beta_{1}EG+\beta_{2}CO_{2}+\beta_{3}TEMP+\beta_{4}TECH+\varepsilon_{it} \end{array}$$

*Model 3*


(3) $$\begin{array}{r} LIFE=\;\beta_{0}+\beta_{1}EG+\beta_{2}CO_{2}+\beta_{3}TEMP+\beta_{4}TECH+\varepsilon_{it} \end{array}$$

Where, *Exposure* represents the exposure to PM2.5, *EG* is the economic growth. *CO*_2_ is the carbon emission from air transport per capita (tons). *TEMP* and *TECH* are Temperature and Environment-related technology, respectively. *Death* and *Life* are Mortality from exposure to ambient PM2.5 (Per 1,000,000) inhabitants and Life expectancy at birth (years).

### Methodology

#### Cross-Sectional Dependence

The analysis of cross-sectional dependence is essential before further analysis of panel data through the CSD test (36). There are chances that spatial or spillover can be between countries' panels, which results in cross-sectional dependence. The basic assumption is the cross-sectional independence of error terms in panels of countries. But there are chances that CSD exits between these panels data models, and inconsistent estimation errors can occur by ignoring this dependence (37).

Following equations is used for CSD test:$CD=\sqrt{\frac{2T}{N\left(N-1\right)}}\left(\overset{N-1}{\underset{i=1}{\sum }}\overset{N}{\underset{j=i+1\rho }{\sum }}ij\right)\to N\left(0,1\right)$

The null hypothesis in the CSD test is no cross-sectional dependence. The distribution of this test is two-tailed N (0,1), where N is ∞ , and T is large.

#### Unit Root Test

Unit root tests are essential to check the stationarity of the data, which is essential for meaningful estimation. There are four types of unit root tests used in literature, including Pesaran and shin, ADF, Phillips-Perron, and LLC tests. In all these tests, the structure of the tests is almost the same, which is as follows:


(4) $$\begin{array}{r} y_{it}=\left(\rho_{i}-1\right)\gamma y_{it-1}+\sum_{j=1}^{Pi}\gamma_{ij}y_{it-j}+\delta_{mi}d_{mt}+V_{it},\;m=1,2,3 \end{array}$$

Where:

*d*_*mt*_= Deterministic component, and if the value of ρ=0, Y has unit root for individual (i). But if ρ <0, then Y is stationary.

In Levin-Lin-(Chao) (LLC) test null hypothesis is presented as *H*_0_:γ = 0, whereas an alternative hypothesis is presented as *H*_*A*_:γ <0. The basic assumption in this test is the similarity of all cross-sections.

In Pesaran and Shin test, null and alternative hypothesis takes the following form:

*H*_0_:γ = 0 (For all values of i)

*H*_*A*_:γ <0 (for at least one i)

In this test, the ADF test is run for each cross-section. Hence, the t-test for γ takes the below-mentioned form.


(5) $$\begin{array}{r} \bar{t}=\frac{1}{N}\overset{N}{\underset{i-1}{\sum }}t_{i}\sim N\left(0,1\right)\text{ if T}\to \infty \text{ followed by N}\to \infty \end{array}$$

In this equation, although T and P_i_ can be different for I, different combinations of these will have different critical values.

#### Pedroni Cointegration

When two or more non-stationary variables have a long-run association, it is called cointegration. Checking cointegration is essential because it enables the researchers to know if there is a stable long-run relationship between the variables of the study. In this study, cointegration test is used where the null hypothesis mentions the non existence of cointegration. The following equation is used to check the cointegration:


(6) $$\begin{array}{r} y_{it}=\alpha_{i}+\delta_{it}+\beta_{i}x_{it}+\varepsilon_{it} \end{array}$$

Where the value of t = 1,….., T and value of I = 1,….., N. T represents the number of observations whereas N. Co-efficient presents a number of panel members presented by β_*i*_, which can be different for each member.

#### ARDL Test

Several econometric techniques can be used to check the long-run association between variables, including fully modified OLS regarding the univariate cointegration (38, 39) and multivariate methodology (40, 41). Although, benefits in terms of less small size bias and provision of more than a single cointegration association makes Johansen approach a valid option. However, the basic requirement for this approach is the same order of integration for all variables.

The Autoregressive Distribution Lag (ARDL) approach presented by Pesaran et al. (42) and Pesaran and Smith (43) is used in this study due to its ability to solve the problems related to the Johansen approach. There are some major advantages of the ARDL approach against other approaches, including the accommodation of serial correlation and endogeneity, hence providing robust results. The same level integration for all variables is not required, but only stationarity of variables is enough in the ARDL method. Also, the ARDL approach can be applied to small sample sizes, and long and short-run associations can be estimated simultaneously through the ARDL method. Due to these advantages, the ARDL approach is best to estimate the relationships of this study.

The ARDL form of Eq (1), (2), and (3) take following form:


(7) $$\begin{array}{l} \Delta Exposure_{t}=\beta_{o}+\beta_{1}Exposure_{,\;t-1}+\beta_{2}EG_{t-1}\\ \;+\beta_{3}CO_{2}{}_{t-1}+\beta_{4}TEMP_{t-1}+\beta_{5}TECH_{t-1}\\ \;+\sum_{k=1}^{n}\gamma_{1k}Exposure_{,\;t-k}+\sum_{k=0}^{n}\gamma_{2k}EG_{t-k}\\ \;+\sum_{k=0}^{n}\gamma_{3k}CO_{2}{}_{t-k}+\sum_{k=0}^{n}\gamma_{4k}TEMP_{t-k}\\ \;+\sum_{k=0}^{n}\gamma_{5k}TECH_{t-k}+Y_{T} \end{array}$$

(8) $$\begin{array}{l} DEATH_{t}=\beta_{o}+\beta_{1}DEATH_{,\;t-1}+\beta_{2}EG_{t-1}+\beta_{3}CO_{2}{}_{t-1}\\ \;+\beta_{4}TEMP_{t-1}+\beta_{5}TECH_{t-1}\\ \;+\sum_{k=1}^{n}\gamma_{1k}Exposure_{,\;t-k}+\sum_{k=0}^{n}\gamma_{2k}EG_{t-k}\\ \;+\sum_{k=0}^{n}\gamma_{3k}CO_{2}{}_{t-k}+\sum_{k=0}^{n}\gamma_{4k}TEMP_{t-k}\\ \;+\sum_{k=0}^{n}\gamma_{5k}TECH_{t-k}+Y_{T} \end{array}$$

(9) $$\begin{array}{l} LIFE_{t}=\beta_{o}+\beta_{1}LIFE_{,\;t-1}+\beta_{2}EG_{t-1}+\beta_{3}CO_{2}{}_{t-1}\\ \;+\beta_{4}TEMP_{t-1}+\beta_{5}TECH_{t-1}\\ \;+\sum_{k=1}^{n}\gamma_{1k}Exposure_{,\;t-k}+\sum_{k=0}^{n}\gamma_{2k}EG_{t-k}\\ \;+\sum_{k=0}^{n}\gamma_{3k}CO_{2}{}_{t-k}+\sum_{k=0}^{n}\gamma_{4k}TEMP_{t-k}\\ \;+\sum_{k=0}^{n}\gamma_{5k}TECH_{t-k}+Y_{T} \end{array}$$

Where, is the difference, *Y*_*T*_ shows error term, β_0_ reflects the constant, β_1_, β_2_ to β_5_ represents the estimation coefficients, γ_1_ to γ_5_ used for dynamics of error correction in ARDL model.

## Results and Discussion

### Preliminary Analysis

Tables 2, 3 presents the results of descriptive statistics and correlation analysis. It can be noted that the highest mean value corresponds to economic growth, whereas the lowest mean value is for temperature. As far as the volatility is concerned, economic growth is highly volatile, whereas life expectancy is least volatile. The results of correlation analysis show that economic growth is not correlated with all public health indicators. However, carbon emission is significantly and negatively correlated with death but significantly and positively correlated with life expectancy. In the case of temperature, it is significantly and positively correlated with exposure and life expectancy but significantly and negatively correlated with death. Technology is positively correlated with exposure only.

**Table 2:** Descriptive statistics.

**Variable**	**Obs**	**Mean**	**Std. Dev**.	**Min**	**Max**
EXPOSURE	192	4.065	0.147	3.681	4.454
DEATH	192	5.859	0.220	5.163	6.371
LIFE	192	4.329	0.023	4.210	4.388
EG	192	12.570	0.848	9.806	14.285
CO_2_	192	7.430	0.733	4.742	8.930
TEMP	192	0.172	0.528	−3.772	0.935
TECH	192	2.734	0.374	1.502	4.320

*EXPOSURE, DEATH, and LIFE represents the Exposure to PM2.5, Mortality from exposure to ambient PM2.5 (Per 1 000 000 inhabitants) and Life expectancy at birth (years). EG mentions the economic growth, CO_2_ is the carbon emission. TEMP and TECH are representing the temperature and technology*.

**Table 3:** Correlation.

	**EXPOSURE**	**DEATH**	**LIFE**	**EG**	**CO2**	**TEMP**	**TECH**
EXPOSURE	1						
DEATH	−0.1633^a^	1					
LIFE	0.3111^a^	−0.5464^a^	1				
EG	−0.0722	0.0362	0.0086	1			
CO_2_	0.0713	−0.3783^a^	0.1599^a^	0.0143	1		
TEMP	0.2880^a^	−0.1809^a^	0.5292^a^	0.2307^a^	−0.0949	1	
TECH	0.2345^a^	−0.0234	0.0714	−0.1790^a^	−0.0836	0.2247^a^	1

*EXPOSURE, DEATH and LIFE represents the Exposure to PM2.5, Mortality from exposure to ambient PM2.5 (Per 1 000 000 inhabitants) and Life expectancy at birth (years). EG mentions the economic growth, CO_2_ is the carbon emission. TEMP and TECH are representing the temperature and technology*.

a *Shows the level of significance*.

#### Cross-Sectional Analysis

There are chances that geographical interdependence exists between the countries under analysis (44, 45). Hence, cross-section dependence can be there. This can be due to spatial and spillover effects besides other unobserved factors. Ignoring this factor of interdependence can lead to biased and inconsistent estimation; hence cross-sectional dependence test is essential. There are different tests to check cross-section dependence, including the LM test (46) and the scaled LM test (47). However, this study uses the Pesaran CD test, and Table 4 presents the results. It is evident that all values are insignificant, showing no cross-section dependence. Hence, the null hypothesis of cross-section independence is accepted, and the alternative hypothesis is rejected.

**Table 4:** Cross-sectional dependence.

**Variable**	**CD test**	***p-*value**
EXPOSURE	0.74	0.458
DEATH	1.22	0.222
LIFE	3.42	0.701
EG	0.64	0.523
CO_2_	0.39	0.693
TEMP	9.06	0.647
TECH	0.63	0.528

*Null hypothesis is cross-sectional independence. The p-values are insignificant, confirming that the null hypothesis is not rejected*.

#### Unit Root and Cointegration Analysis

The results regarding the panel unit root tests are presented in Table 5. Two unit root tests are used, including the Im-Pesaran-Shin unit root test and the LLC unit root test. The Im-Pesaran-Shin unit root test shows that all variables except carbon emission are stationary at level, but all variables are stationary at first. In the case of the LLC unit root test, the majority of variables have unit root at level, but at first, no unit root is present regarding all variables. Hence, it can be said that at the first difference, all variables in the panel series have no unit root. The integration of variables in the panel series is I (1) order.

**Table 5:** Unit root test.

	**Im-Pesaran-Shin unit-root test**			
**Variables**	**At level**	***p-*value**		**A difference**	***p*-value**
EXPOSURE	−2.775 ^a^	0.003		−10.034^a^	0.000
DEATH	−2.987^a^	0.001		−9.077^a^	0.000
LIFE	−4.672^a^	0.000		−11.345^a^	0.000
EG	−4.359 ^a^	0.000		−10.502^a^	0.000
CO2	0.563	0.713		−4.422^a^	0.000
TEMP	−5.651^a^	0.000		−12.350^a^	0.000
TECH	−8.505^a^	0.000		−8.437^a^	0.000
LLC unit root test
EXPOSURE	−1.935^b^	0.026		−7.489^a^	0.000
DEATH	−0.615	0.269		−5.322^a^	0.000
LIFE	−1.095	0.137		−11.345^a^	0.000
EG	−1.282	0.100		−4.568^a^	0.000
CO2	0.317	0.625		−5.579^a^	0.000
TEMP	−2.753^a^	0.003		−6.857^a^	0.000
TECH	−4.795^a^	0.000		−8.613^a^	0.000

*EXPOSURE, DEATH, and LIFE represents the Exposure to PM2.5, Mortality from exposure to ambient PM2.5 (Per 1,000,000 inhabitants) and Life expectancy at birth (years). EG mentions the economic growth, CO_2_ is the carbon emission. TEMP and TECH are representing the temperature and technology*.

a, b * mentions the level of significance at 1% and 5%, respectively*.

As all variables are stationary at a level, exploring the cointegration between variables is necessary. Pedroni cointegration is used in this study to check the long-run association between variables, as followed by Sarwar et al. (48) and Waheed et al. (49), and results are reported in Table 6. It can be noted that six out of seven statistics are significant at a 1% level of significance. This indicates that the null hypothesis of no cointegration is rejected and there is a long-run relationship between the study variables.

**Table 6:** Pedroni cointegration.

	**Model 1**		**Model 2**		**Model 3**	
	**Stat**	**Weighted stat**	**Stat**	**Weighted stat**	**Stat**	**Weighted stat**
Panel v-Statistic	−0.781	−0.537	−0.679	−1.134	−0.966	−2.415
Panel rho-Statistic	−3.990^a^	−3.702^a^	−3.714^a^	−3.319^a^	−3.601^a^	−3.970^a^
Panel PP-Statistic	−8.767^a^	−8.656^a^	−9.784^a^	−11.356^a^	−10.35^a^	−13.075^a^
Panel ADF-Statistic	−3.689^a^	−3.286^a^	−4.346^a^	−4.488^a^	−4.72^a^	−4.573^a^
Group rho-Statistic	−3.345^a^		−3.167^a^		−3.340^a^	
Group PP-Statistic	−13.745^a^		−15.279^a^		−14.77^a^	
Group ADF-Statistic	−4.246^a^		−4.741^a^		−4.602^a^	

a *shows the level of significance at 1%*.

#### ARLD Estimation

##### Long-Run Analysis

Cointegration tests suggests that the ARDL approach is appropriate to check the long-run and short-run association between variables. Hence, the ARDL approach is used for estimation, and Table 7 shows the results. In the long run, it can be seen that in all models, economic growth is significantly and positively associated with public health at a 5% level of significance in Model 1 and 3 and a 1% level of significance in Model 2. Hence, it can be said that economic growth increases exposure to PM2.5 and mortality but enhances the life expectancy. Zheng et al. (50) also found that economic growth can increase exposure to PM2.5. But results regarding the economic growth and mortality are against the findings of Dadgar and Norström (51). The reason could be the train of GCC countries, industrial development of GCC countries in recent years, which causes the higher PM2.5. This leads to a high mortality rate. The result regarding the economic growth and life expectancy is consistent with the findings of Niu et al. (17). This positive association is due to prioritizing the improvement in the healthcare sector in GCC countries. The spending on the healthcare sector in GCC is about US$ 104.6 billion currently, which is 6.6% higher since 2017.

**Table 7:** ARDL estimation.

**Long run**	**Model 1**	**Model 2**	**Model 3**
EG	0.033^b^	0.433^a^	0.091^b^
CO_2_	0.041^a^	0.121^a^	0.015^a^
TEMP	0.271^a^	−0.212	0.064^a^
TECH	0.107^a^	0.037	0.003
**Short-run**
D EG	0.029	−0.052	0.098
D CO_2_	−0.034	0.002	−0.004
D TEMP	−0.008	−0.023	0.006
D TECH	0.018	−0.003	0.004
C	1.072^a^	−0.132	1.537^a^
ECT	−0.361^a^	−0.197^c^	−0.366^a^

*EXPOSURE, DEATH and LIFE represents the Exposure to PM2.5, Mortality from exposure to ambient PM2.5 (Per 1,000,000 inhabitants) and Life expectancy at birth (years). EG mentions the economic growth, CO_2_ is the carbon emission. TEMP and TECH are representing the temperature and technology. C and ECT are constant and error correction terms respectively*.

a, b, c *Mentions the significance level at 1% and 5%, respectively*.

The coefficient of carbon emission is positive and significant at a 1% level of significance in all models. It can be seen that carbon emission increases exposure to PM2.5 also, suggest that carbon emission is the main culprit behind increased PM2.5 exposure. Likewise, carbon emission is also significantly and positively related to mortality, consistent with the findings. However, carbon emission is significantly and positively related to life expectancy showing that carbon emission enhances life expectancy. This result contrasts with many previous studies (17, 29, 30). One possible explanation of this positive association is improvement in overall health is explained by Gangadharan and Valenzuela (52), who assert that healthcare gains cancel the environmental losses due to improvement in income level. The same is true for GCC countries where the income level is improved significantly, which improves the quality of health. Due to improvement in the standard of living through high income, life expectancy improves despite increased carbon emission. Also, a high-income level enables the residents of these countries to get better treatment from foreign countries, including the US and Europe and this also improves life expectancy.

In the case of temperature, the coefficient is positive and significant in Model 1 and 3 at a 1% significance level. This suggests that temperature enhances the exposure to PM2.5 but improves life expectancy, which is against the findings of Yang et al. (2), Ostro et al. (6) and Gasparrini et al. (23). The reason could be the adaptive behavior of people in GCC countries. The climate of these countries is inherently hot; hence people get accustomed to hot weather. Also, adaptive behavior, including clothing and staying indoor, reduces the harmful impacts of temperature, including mortality. Likewise, modified and effective cooling devices are installed at homes and offices now due to improvement in income level, which protects the population from harmful impacts of high temperature. Hence, besides an increase in temperature, life expectancy tends to improve.

Technology is significantly and positively related to public health in only Model 1 at a 1% significance level. This result is against the findings of Jiang et al. (26) and Hanzl (53), who suggest that technological improvements in the environment enhance public health. Efforts are made in GCC countries to change the overall energy mix by including renewable sources. But still, fossil fuels are the major source of energy. The industrial and transportation sector is dependent on fossil fuels, leading to a high concentration of PM2.5 in the atmosphere (1) despite making technical progress related to the environment.

##### Short-Run Analysis

In the short run, all variables in all models are insignificantly related to public health. Although in one model, economic growth is negatively related to public health. In contrast, carbon emission is negatively related in two models, which shows that carbon emission can reduce public health in the short run. The same is true for temperature because temperature proves to be a public health issue in the two models. Technology is positively related to the majority of the models showing that environmental technology can enhance public health. The contributing factors regarding public health are not shown to be important in the short run.

#### Dumitrescu-Hurlin Panel Causality

Table 8 presents the results of the Pairwise Dumitrescu-Hurlin panel causality test, which is the advanced version of the Granger test, to check if causality is present between study variables. It can be seen that there is causality between economic growth and public health because, in the majority of models, the Zbar value is significant. The causality from carbon emission to public health is present due to significant Zbar statistics, but there is no causality from public health to carbon emission. In the case of temperature and public health, no causal relationship is there. The same is true for technology and public health.

**Table 8:** Pairwise Dumitrescu-Hurlin panel causality test.

	**Model 1**	**Model 2**	**Model 3**
	**Zbar-stat**	**Zbar-stat**	**Zbar-stat**
EG does not homogeneously cause PH	1.273	−0.226^b^	4.252^a^
PH does not homogeneously cause EG	5.529^a^	2.949^a^	1.067
CO_2_ does not homogeneously cause PH	0.859^a^	−0.725^a^	1.835 ^c^
PH does not homogeneously cause CO_2_	6.411	6.264	−0.889
TEMP does not homogeneously cause PH	−1.537	−0.713	−0.789
PH does not homogeneously cause TEMP	0.388	0.131	−0.773
TECH does not homogeneously cause PH	−1.138	−0.064	−0.406
PH does not homogeneously cause TECH	−0.772	0.139	0.504

*EXPOSURE, DEATH and LIFE represents the Exposure to PM2.5, Mortality from exposure to ambient PM2.5 (Per 1 000 000 inhabitants) and Life expectancy at birth (years). EG mentions the economic growth, CO_2_ is the carbon emission. TEMP and TECH are representing the temperature and technology*.

a, b, c *Mentions the level of significance at 1% and 5%, respectively*.

## Conclusion

The current study aims to explore the determinants of public health in GCC countries from 1990 to 2020. After conducting the unit root and cointegration test, the ARDL approach explores the long-run and short-run association between variables. It is noted that all variables are significantly related to public health only in the long run. Economic growth proved to be an enhancer of exposure to PM2.5 and mortality, improving life expectancy. Also, carbon emission is significantly and positively related to all public health measures. Carbon emission enhances PM2.5 exposures and increases the mortality rate. But more carbon emission results in high life expectancy. Likewise, temperature and environmental technology also add to GCC countries' public health measures.

In light of these findings, some major policy implications are as follows: the government should try to improve the quality of treatment available in the country. When people travel abroad for treatment, healthcare costs increase as authorities of government agencies pay this cost. A new economic development pattern should be followed to reduce the harmful impacts of economic growth on health. Decelerating economic growth is not an option, but the link between PM2.5 concentration and economic growth should be decoupled. To do this, an intensive growth pattern should be followed instead of an extensive pattern. Policymakers should introduce environmental policies to reduce the risk related to air pollution due to economic growth. Policymakers should divert investment toward research and development efforts to introduce upgraded and environmentally friendly products, including cooling devices. It will reduce the harmful impacts of carbon emission and help cope with the increasing temperature of the region. Healthcare spending can also be reduced by reducing imported and branded products. Value-added devices and generic drugs are used in developed countries, but GCC countries miss this, increasing overall health expenditure. Revising the overall energy mix is also a valid option to reduce the harmful impacts of increasing heat and carbon emission. In this regard, using renewable energy sources, including wind and solar power, will reduce health-related issues.

## Data Availability Statement

Publicly available datasets were analyzed in this study. This data can be found here: world bank.

## Author Contributions

GA has form the idea and did the testing. MNK did the writing task. MSK supervised and revised the manuscript. All authors contributed to the article and approved the submitted version.

## Conflict of Interest

The authors declare that the research was conducted in the absence of any commercial or financial relationships that could be construed as a potential conflict of interest.

## Publisher's Note

All claims expressed in this article are solely those of the authors and do not necessarily represent those of their affiliated organizations, or those of the publisher, the editors and the reviewers. Any product that may be evaluated in this article, or claim that may be made by its manufacturer, is not guaranteed or endorsed by the publisher.
